# A Case–Control Study of Socio-Economic and Nutritional Characteristics as Determinants of Dental Caries in Different Age Groups, Considered as Public Health Problem: Data from NHANES 2013–2014

**DOI:** 10.3390/ijerph15050957

**Published:** 2018-05-10

**Authors:** Laura A. Zanella-Calzada, Carlos E. Galván-Tejada, Nubia M. Chávez-Lamas, Ma. del Carmen Gracia-Cortés, Arturo Moreno-Báez, Jose G. Arceo-Olague, Jose M. Celaya-Padilla, Jorge I. Galván-Tejada, Hamurabi Gamboa-Rosales

**Affiliations:** 1 Unidad Académica de Ingeniería Eléctrica, Universidad Autónoma de Zacatecas, Jardín Juarez 147, Centro, Zacatecas 98000, Zac, Mexico; lzanellac@uaz.edu.mx (L.A.Z.-C.); morenob20@uaz.edu.mx (A.M.-B.); arceojg@uaz.edu.mx (J.G.A.-O.); gatejo@uaz.edu.mx (J.I.G.-T.); hamurabigr@uaz.edu.mx (H.G.-R.); 2 Clínica Comunitaria de Tacoaleche, Unidad Académica de Odontología, Universidad Autónoma de Zacatecas, Jardín Juarez 147, Centro, Zacatecas 98000, Zac, Mexico; nubiachavez@uaz.edu.mx (N.M.C.-L.); gacc005340@uaz.edu.mx (M.d.C.G.-C.); 3 CONACYT, Universidad Autónoma de Zacatecas, Jardín Juarez 147, Centro, Zacatecas 98000, Zac, Mexico; jose.celaya@uaz.edu.mx

**Keywords:** NHANES, dental caries, fast backward variable selection, net reclassification improvement, computer-aided diagnosis, statistical analysis, predictive multivariate models

## Abstract

One of the principal conditions that affects oral health worldwide is dental caries, occurring in about 90% of the global population. This pathology has been considered a challenge because of its high prevalence, besides being a chronic but preventable disease which can be caused by a series of different demographic, dietary, among others. Based on this problem, in this research a demographic and dietary features analysis is performed for the classification of subjects according to their oral health status based on caries, according to the age group where the population belongs, using as feature selector a technique based on fast backward selection (FBS) approach for the development of three predictive models, one for each age range (group 1: 10–19; group 2: 20–59; group 3: 60 or more years old). As validation, a net reclassification improvement (NRI), AUC, ROC, and OR values are used to evaluate their classification accuracy. We analyzed 189 demographic and dietary features from National Health and Nutrition Examination Survey (NHANES) 2013–2014. Each model obtained statistically significant results for most features and narrow OR confidence intervals. Age group 2 obtained a mean NRI = −0.080 and AUC = 0.933; age group 3 obtained a mean NRI = −0.024 and AUC = 0.787; and age group 4 obtained a mean NRI = −0.129 and AUC = 0.735. Based on these results, it is concluded that these specific demographic and dietary features are significant determinants for estimating the oral health status in patients based on their likelihood of developing caries, and the age group could imply different risk factors for subjects.

## 1. Introduction

Health is a condition that presents difficulties in its description due to its different definitions. According to the World Health Organization (WHO), health can be defined as a physical, mental, and social healthy status and not only as the absence of diseases. Quality of life is included, defined as an individual perception of life position, in the context of the cultural environment and the individual’s relationships with the objectives, expectations, standards, and concerns in life. Due to this concept, there are some factors that affect the health of subjects. These factors can be evaluated through three different dimensions: general (lifestyle), particular (life conditions), and singular (type of life) [1,2].

Oral health is considered to be an integral and essential part of health, and can compromise the quality of life of all groups of people, including infants, adolescents, young people, adults, and older adults without distinguishing between age; sex; social level; culture; economic, educational, and marital status; previous caries history; current caries index; levels of microbiological factors; drawing of family caries; and the environment in which they are found [1,2,3].

These characteristics have supported and facilitated the recognition of different determinants that contribute to negatively modifying oral health status, since its appearance depends on the conjugation of biological factors such as dental anatomy, diet consumption, and the appearance of oral diseases, where the relationship between dietary and demographic features and the appearance of dental caries is widely demonstrated [4,5,6,7,8,9]. In oral public health, dental caries are a challenge because it is a condition that represents a chronic and preventable disease, affecting around 90% of the global population [8], becoming an oral health problem. In the early stages of life, teeth usually do not present problems related to this disease, but throughout life, dental caries can be developed by different factors such as those mentioned above, being the main reason for the attention of such conditions [9].

Due to the difficulty of controlling the incidence of caries, some studies have been developed that have implemented algorithms and performed analysis based on computer-aided diagnosis (CADx) in order to provide an automated tool for the detection of caries based on different types of features, with the aim of obtaining a better prognosis for patients and identifying the risk factors that can contribute to their prevention. One of the main CADx tools that has been used for the detection of diseases is the net reclassification index (NRI), which is a measure for evaluating the improvement in the prediction performance of the disease gained by adding a marker to baseline predictors. Other statistical parameters that are commonly used in clinical research include the receiver operating characteristic (ROC) curve, the area under the curve (AUC), the benefit function, and the Brier score, among others [10].

Based on this general oral health problem description, the main contribution of this paper is to analyze the relationship between demographic and dietary features and the dental caries status, in order to develop a multivariate model (based on CADx) for three different age groups for the classification of subjects according to their dental caries status (presence/absence), in addition to looking for the different features that affect the age groups. These multivariate models were developed through a statistical approach using fast backward selection (FBS) for the feature selection, NRI for the validation, in order to know the reclassification proportion of subjects, and the ROC curve, which allows the sensitivity (true positives) and specificity (true negatives) of the model’s classification to be obtained.

According to the contributions mentioned above, the hypothesis of this work makes reference to the possibility of developing a multivariate model through statistical analysis—based on demographic and dietary features that were provided from National Health and Nutrition Examination Survey (NHANES) 2013–2014—that is able to automatically classify between patients with the presence and absence of caries, in order to find a tool that provides information about the risk factors that make subjects vulnerable to dental caries, according to their age range.

### Related Work

There are several approaches to identifying caries risk determinants. In the work of Lam C. et al. [11], it was found that dental visits, brushing frequency, lower parental perceived importance of baby teeth, and weaning onto solids are determinants associated with plaque accumulation; however, they only validated these factors in children. On the other hand, I. B. Fernandes et al. [12] presented a cross-sectional study of 274 children and their mothers based on demographic/socio-economic status; they used a Poisson regression to perform the analysis, finding that dental caries can be mainly found in mothers of children aged 1, which demonstrates a relationship between demographic/socio-economic status and caries with children. Other types of risk factors are associated with genetic and dietary data, like in the work of A. Lips et al. [13], where the association between genetic polymorphisms and risk of dental caries was demonstrated for most of the salivary proteins. Diabetic and hypertensive patients have dietary risk factors that can lead to oral health problems, demonstrated by Asif Ahmed et al. [14] through a statistical analysis using clinical data, aiming to identify that patients with oro-dental problems were hemodynamically stressed.

This paper is organized as follows. Section 2 provides a description of the data set used for this research, the statistical methods performed, as well as the experimentation conditions. The results obtained from the statistical analysis are presented in Section 3. Discussion and conclusions are described in Section 4, and finally, future work is briefly mentioned in Section 5.

## 2. Materials and Methods

The development of this work was performed using the data from the National Health and Nutrition Examination Survey (NHANES, 2013–2014). These data are described in this section, as well as the patient selection, data preprocessing, and methods for the acquisition of models.

### 2.1. Study Design

**Figure 1:**
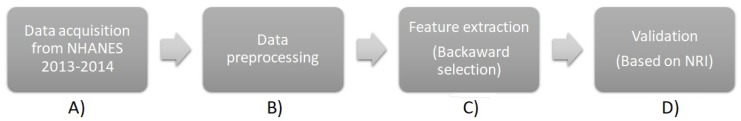
Flowchart of the steps followed in methodology. NHANES: National Health and Nutrition Examination Survey; NRI: net reclassification index.

The study design of this work is presented in Figure 1, beginning with the data acquisition from NHANES 2013–2014 in (A), selecting three different types of features from the public datasets: dietary, demographic, and examination. A brief data preprocessing is carried out in (B), applying different techniques to solve the missing data problem and to remove any singular value presented in features, and to separate the data in three different datasets, according to the age range of the participants. Then, the feature selection is presented in (C), which is performed using the statistical technique FBS, obtaining three multivariate models (one for each dataset) containing the most statistically significant features. Finally, a validation process for each multivariate model was performed using the NRI technique in addition to the statistical parameters AUC, ROC curve, and OR, in order to evaluate the model’s accuracy.

### 2.2. Setting

For this work, the public data of the NHANES program were used. NHANES collects a repository of information from US participants based on a series of different questionnaires in order to obtain data for health status knowledge. The data used were from the 2013–2014 period, and from three different types of questionnaires: dietary, demographic, and examination.

### 2.3. Dataset Description

NHANES is a federal agency that produces data and materials for the public domain from the health and nutritional status of adults and children in the United States, including ethnic groups. The survey combines interviews and physical examinations, allowing the development of studies using the clinical, para-clinical, and demographic information of subjects. NHANES is an initiative that was founded by the Centers for Disease Control and Prevention (CDC) and the National Center for Health Statistics (NCHS) [15].

The main objectives of NHANES are to estimate the number and percentage of persons in the US population with selected diseases and risk factors; to monitor trends in the prevalence, awareness, treatment, and control of selected diseases; to monitor trends in risk behaviors and environmental exposures; to study the relationship between diet, nutrition, and health; to explore emerging public health issues and new technologies; and to provide baseline health characteristics that can be linked to mortality data from the National Death Index or other administrative records.

NHANES data include a series of different types of characteristics, which are called features in this work; among them are included demographic data (individual, family, and household level information), dietary data (total nutrient intake), and health-related information (public health significance in areas of surveillance, prevention, treatment, dental care utilization, health policy, and evaluation of Federal health programs). A health-related component that is critical to this study is the oral health examination, which is carried out by trained medical personnel and is related to the presence or absence of dental caries.

#### 2.3.1. Participants

The content of these features was obtained from subjects that were submitted to a series of different questionnaires, related to the different features. These subjects were women and men belonging to different counties in the USA, divided into 15 groups based on their characteristics, and were randomly selected with a computer algorithm by NHANES. The algorithm consists of a complex multistage probability design to choose the participants, with a series of stages. The first selection is related to primary sampling units (PSUs), which are counties or small groups of them; the next selection is about segments within PSUs that constitute a group of households; then, a selection of specific households is performed; and finally, a selection of individuals within a household.

#### 2.3.2. Variables

The data used are a collection of 190 features (described in Appendix A). From these features, 189 correspond to demographic and dietary data, which were used as descriptors or inputs. Demographic data refers to individual-, family-, and household-level information, while dietary data refers to the total nutrient intake. The remaining feature was contained by the dental caries status, and it was set as output. This output feature describes a patient’s dentition; if the subject has suffered from caries or restorations they are assigned the value “1”, while if the subject has not, they are assigned the value “0”.

### 2.4. Statistical Methods

All data preprocessing, analysis, and validation were carried out using the free software *R* (version 3.4), which is an environment for statistical computing and graphics that provides tools for different data mining techniques [16]. Packages for *R* that were used are rms (version 5.1.2) [17], MASS (version 7.3.49) [18], rminer (version 1.4.2) [19], pROC (version 1.10.0) [20], randomForest (version 4.6.12) [21], and nricens (version 1.5) [22].

#### 2.4.1. Data Preprocessing

The first preprocessing step consisted of manually removing those input features that presented a high percentage of missing data (≥70%) or singular values, which made their use impossible for the statistical analysis of this work. Missing data were represented as Not a Number (NaN), while singular features presented the same value in all rows of a column or presented multiple values with another feature between each other.

The remaining input features presented <20% of missing values, and were imputed using the rfimput function from the *R* package randomForest. The imputation based on this function consists of replacing all NaN with the median value of the column where the missing data are located [21].

The next part in the preprocessing step consisted of dividing the database in three different datasets, where subjects were classified into three different age groups, according to the age range where they belong. The age ranges were defined by the contribution of Dr. Nubia M. Chávez-Lamas, who is an expert in oral health and an author of this work.

Finally, after the classification of subjects in the age groups, it was necessary to repeat the process of removing all features that presented singular values for group 2 (10–19 years), due to the poor quantity of data that were contained within it. Those features were removed because they presented the same value in all their rows, which means that all subjects presented the same information for those features, being useless for this analysis, where we are looking for the most relevant differences that subjects present. For age groups 3 and 4 it was not necessary to remove features due to singular values.

#### 2.4.2. Feature Selection

After all data were subjected to a preprocessing step, three different datasets were obtained containing subjects belonging to the three different age ranges. Then, each dataset was subjected separately to a feature selection process using an FBS approach in order to select the features that presented the best performance for the classification of patients.

The FBS process consisted of initially subjecting all the features to a multivariate logistic regression (LR), in order to obtain a general model based on the relationship between input features and the output feature.

LR is an analysis that consists of a statistical technique to model the relationship between the input/independent features and the output/dependent feature, using binary data. It measures the contribution of different factors to the occurrence of a simple event, and its main objective is to model the influence of the probability of this event. In Equation (1), the simplest representation of a model obtained by this method is presented, where *y* is the output feature. *y* must be subjected to a logarithmic transformation, represented as logit, because the model is initially exponential. Through this transformation, it is possible to use it as a lineal function; *w* is an offset term that can be included, and is also known as bias; $\beta_{1}\ldots \beta_{n}$ are the slopes or coefficients calculated to obtain the real solution of the equation, and $x_{1}\ldots x_{n}$ are the input features to be analyzed. Models can be composed by the number of input features needed [23].

(1) $$logit\left(y=1\right)=w+\beta_{1}x_{1}++\beta_{2}x_{2}+\ldots +\beta_{n}x_{n}$$

Then, after applying LR, a feature selection process was carried out using the fbw function from the rms package, which performs a numerically stable version of FBS, using a method based on Lawless and Singhal [24].

FBS belongs to the stepwise methods that are used for feature selection, which are techniques that add and/or delete features through iterations, keeping only those features that present the best aptitude in the required task. This method starts subjecting the model, contained by all *n* features, to multiple regressions, and eliminating features one at a time. At each step, the feature that was selected to be deleted presented the least inflation in the residual sum of squares. The iterative technique continues until only one feature is left in the model or until a stopping rule or threshold is satisfied. For this work, the stopping rule selected was the *p*-value because it is one of the main parameters used for this technique as a threshold, according to different works [25,26].

The *p*-value or the observance significance level is the smallest value obtained where the null hypothesis can be rejected and is calculated using the sampling distribution of the test statistic under the null hypothesis [2].

In FBS, it is necessary to be in a situation where the number of observations is greater than the number of features, in order to avoid problems of overfitting. It is important to mention that the threshold that was selected for the feature selection in age group 2 (*p*-value < 0.5) was different from the selected for the age groups 3 (20–59 years) and 4 (60 or more years) (*p*-value < 0.005). This difference between age groups was due to the significant difference in the number of subjects and features that were contained in the age groups, being specially remarkable in age group 2, causing difficulties in finding a general model for this group.

Finally, a multivariate model was developed for each age group. These multivariate models were contained by a series of demographic and dietary features that were selected due to their significant performance in the classification of subjects with the presence or absence of caries.

After this step, all models were subjected to a validation process based on an NRI approach and an analysis of sensitivity (true positives) and specificity (true negatives).

#### 2.4.3. Validation

NRI is a very popular measure that was introduced in 2008 as a new statistic for evaluating the performance of prediction models based on a cross-validation process. This evaluation is carried by adding a marker to a set of reference predictors to predict a binary outcome, and it is based on the principle on comparing if a model *B* can predict an outcome better than a model *A*. One of the main purposes of the use of prediction models is to assess whether the addition of a new prediction feature improves the discrimination of who will experience an event from those who will not. A very simple measure that is used to validate the discrimination capacity of the models is to calculate the difference between the average of the predicted risk obtained in those that have a value of the binary result and those who have the other value; the greater this difference, the better the discrimination.

NRI is a technique that has demonstrated clinical utility based on the improvement of clinical decision-making that a specific model can achieve. Through a statistical validation (comparing a first model vs. a second model), NRI measures the proportion of subjects with the condition that is correctly reclassified up to high risk and incorrectly reclassified down to low risk; and on the other hand, the proportion of subjects without the condition that is correctly reclassified down to low risk and incorrectly reclassified up to high risk. This validation process is a confounder-adjusted estimate that obtains as result the reclassification value and the proportion rates, which represent a confidence interval of the precision [10,22].

For this work, NRI was performed using the nricens package, which provides the functions to estimate the NRI parameters for risk prediction models with two main approaches: time to event, and binary response data. The binary response data approach was selected, which can be calculated with the nribin function. The risk category can be calculated using LR. Additionally, confidence intervals were calculated using the percentile bootstrap method. For the risk category calculation, it was necessary to specify a cut parameter, which represents the cutoff values of the risk category. Then, from this function a parameter was returned with its confidence intervals (i.e., the point and the intervals estimated by the NRI process and its components). The up and down values were also returned, which are logical values that allow the determination of the number of subjects that belong to UP (high risk) and DOWN (low risk) parameters, with their respective reclassification tables of objects that correspond to all, case, and control subjects [22].

NRI has gained great popularity in a series of biomedical applications; nevertheless, it has been demonstrated that some problems related to the fitting of the risk models may be present. Based on this caution, the results obtained under NRI are backed up with the statistical parameters OR, AUC, and ROC curve [27].

The OR parameter is defined as the value of the relationship between an input feature and an outcome, and it is represented as the probability that this specific outcome will occur under a particular feature, against the probability that the outcome will occur with the absence of that feature. Additionally, the 95% confidence intervals (from 2.5% to 97.5%) are calculated [28]. Equation (2) represents the calculation of OR, where $P_{a}$ is the probability of a first event occurring, while $P_{b}$ is the probability of a second event occurring, taking into account that the first one has already occurred.

(2) $$ODDSratio=\frac{\frac{P_{a}}{1-P_{a}}}{\frac{P_{b}}{1-P_{b}}}$$

The AUC value is a standard method that evaluates the accuracy of the classification model based on the relationship between the specificity and the sensitivity, obtained by calculating the ROC curve [29].

Sensitivity is defined as the proportion of subjects with one condition that were classified as positive; this value is calculated by Equation (3), where TP represents the number of true positives and FP represents the number of false positives.

(3) $$PPV=\frac{TP}{TP+FP}$$

Specificity is defined as the proportion of subjects without a condition that were classified as negative; this value is calculated by Equation (4), where TN represents the number of true negatives and FN represents the number of false negatives.

(4) $$NPV=\frac{TN}{TN+FN}$$

## 3. Results

Results obtained from the feature extraction using a FBS approach for each dataset (corresponding to the three different age groups) and the validation of each multivariate model using NRI, OR, ROC, and AUC are presented in this section.

### 3.1. Participants

**Figure 2:**
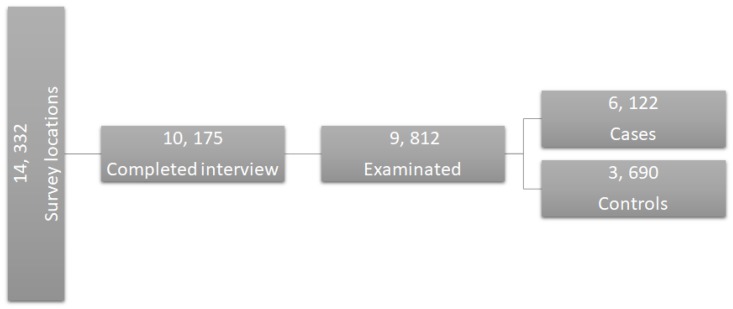
Subject selection process.

The subject selection process is shown in Figure 2, where from the total 14,332 persons that were selected from the different survey locations, 10,175 completed the interview and 9812 were examined. From the 9812 total subjects, 6122 belonged to cases and 3690 belonged to controls. Case subjects were those that presented dental caries before or during the interviews, while control subjects were those that did not present dental caries before or during the interviews. The number of women were 4831 and the number of were men 4982.

#### Descriptive Data

The main target population for NHANES is the non-institutionalized civilian resident population of the USA. There have been a series of changes in the design of the population selection in order to sample larger numbers of specific subgroups that present particular public health characteristics, thus to increase the reliability and precision of health status indicators. NHANES started these design changes in 2011, including in its population the oversampled subgroups survey cycle:

- Hispanic persons;
- Non-Hispanic black persons;
- Non-Hispanic Asian persons;
- Non-Hispanic white and other* persons at or below 130% of the poverty level; and
- Non-Hispanic white and other* persons aged 80 years and older.

### 3.2. Outcome Data and Main Results

Here are reported the preprocessing, statistical analysis, and validation steps. For the preprocessing are presented the number of features that were removed due to missing data and singular values, in addition to the imputation technique of the remaining missing data. Then, for the statistical analysis are presented the multivariate models that were developed using the FBS method, including all features for each model and their description. Finally, the validation results are shown, presenting the values obtained for the NRI technique and the values obtained for the AUC and OR, besides the ROC curve.

#### 3.2.1. Preprocessing

The first step of the statistical analysis that was performed was a preprocessing. The first part of the preprocessing consisted of manually removing 85 features from the 189 total input features because they presented a high percentage of missing data or singular values. Then, from the 104 remaining input features, there were some data that presented <20% of missing values, which were imputed using the rfimput function from the *R* package randomForest.

Once all data were complete, they were separated in three different datasets according to the age group where the population belonged. The age groups and the number of subjects that were contained in each group are presented in Table 1. Age group 1 was discarded from the analysis of this work because it was not contained by any subject. Therefore, only age groups 2, 3, and 4 were retained. Finally, the process of removing all features that presented singular values for age group 2 was repeated, eliminating 48 features and keeping 56. For age groups 3 and 4 it was not necessary to remove features due to singular values.

#### 3.2.2. Feature Selection and Validation

After the preprocessing step, a feature selection was performed in order to obtain a multivariate model for each age group containing the most statistically significant features from the total features set.

The multivariate model obtained for age group 2 contained 33 features, that obtained for age group 3 contained 18 features, and that obtained for age group 4 contained 10 features, out of a total of 104 features.

Table 2 presents the 33 features contained in the model obtained for age group 2, with their respective description, OR values, and confidence intervals.

**Figure 3:**
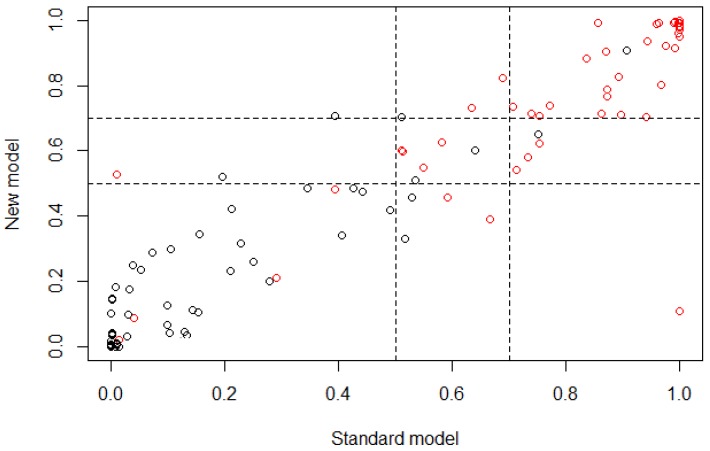
Plot of the risk category based on the NRI calculus for age group 2; horizontal axis represents the standard model or the multivariate model before the feature selection and vertical axis represents the new model or the multivariate model after the feature selection (control ∘, case ∘).

After the validation process using NRI for age group 2, the graph from Figure 3 was obtained, where the risk category under the LR technique is presented, defining as cutoff the range from 0.5 to 0.7. Subjects obtaining a classification probability >0.5 belong to controls and subjects obtaining a classification probability ≥0.7 belong to cases. The NRI values obtained were NRI=−0.080, +NRI=−0.080, −NIR=0, with the proportion rates Pr(Up|Case)=0.040, Pr(Down|Case)=0.120, Pr(Down|Control)=0.048, Pr(Up|Control)=0.048.

Table 3 presents three reclassification tables, measuring the true and false positives and the true and false negatives by comparing the results obtained in the classification of subjects using the standard model or the model before the feature selection and the new model generated through the feature selection for age group 2.

Table 4 presents the 18 features that were contained in the model obtained for age group 3, with their respective description, OR values, and confidence intervals.

**Figure 4:**
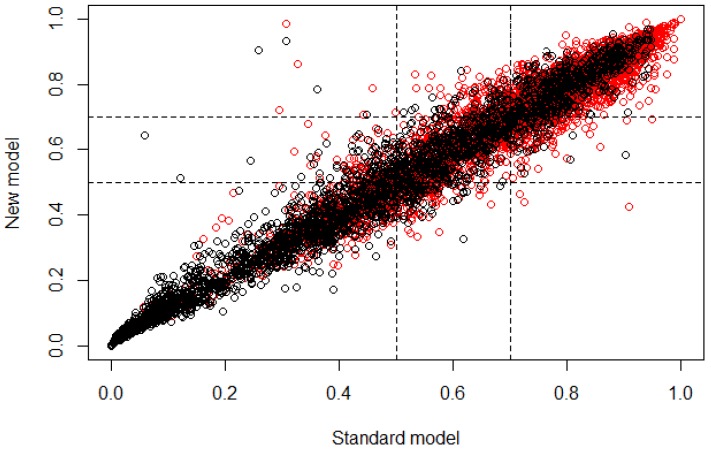
Plot of the risk category based on the NRI calculus for age group 3. The horizontal axis represents the standard model or the multivariate model before the feature selection and the vertical axis represents the new model or the multivariate model after the feature selection (control ∘, case ∘).

Then, after the validation process using NRI for age group 3, the graph in Figure 4 was obtained, where the risk category is presented, defining the range from 0.5 to 0.7 as cutoff, where subjects that obtain a classification probability >0.5 belong to controls and subjects that obtain a classification probability ≥0.7 belong to cases. The NRI values obtained were NRI=−0.024, +NRI=−0.019, −NIR=−0.005; with the proportion rates Pr(Up|Case)=0.055, Pr(Down|Case)=0.074, Pr(Down|Control)=0.055, Pr(Up|Control)=0.060.

Table 5 presents three reclassification tables, measuring the true and false positives as well as the true and false negatives by comparing the results obtained in the classification of subjects using the standard model or the model before the feature selection and the new model generated through the feature selection for age group 3.

Table 6 presents the 10 features that were contained in the model obtained for age group 4, with their respective description, OR values, and confidence intervals.

**Figure 5:**
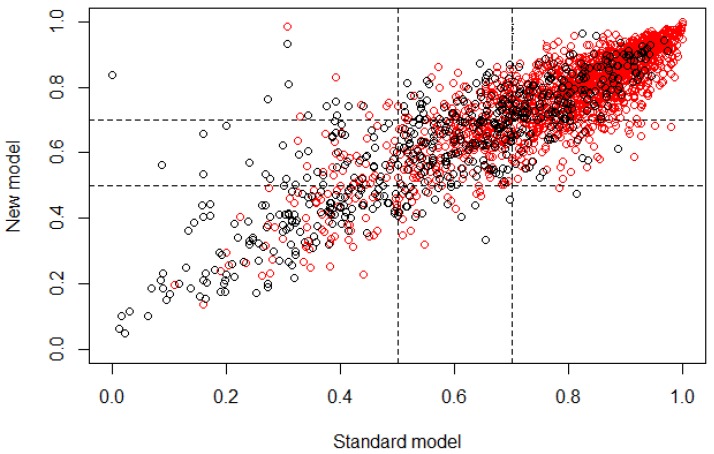
Plot of the risk category based on the NRI calculus for age group 4. The horizontal axis represents the standard model or the multivariate model before the feature selection, and the vertical axis represents the new model or the multivariate model after the feature selection (control ∘, case ∘).

Then, after the validation process using NRI for age group 4, the graph from Figure 5 was obtained, presenting the risk category, defining as cutoff the range from 0.5 to 0.7, where subjects that obtain a classification probability >0.5 belong to controls and subjects that obtain a classification probability ≥0.7 belong to cases. The NRI values obtained were NRI=−0.129, +NRI=−0.013, −NIR=−0.116. The proportion rates were Pr(Up|Case)=0.083, Pr(Down|Case)=0.096, Pr(Down|Control)=0.081, Pr(Up|Control)=0.197.

Table 7 presents three reclassification tables, measuring the true and false positives as well as the true and false negatives by comparing the results obtained in the classification of subjects using the standard model or the model before the feature selection and the new model generated by the feature selection for age group 4.

**Figure 6:**
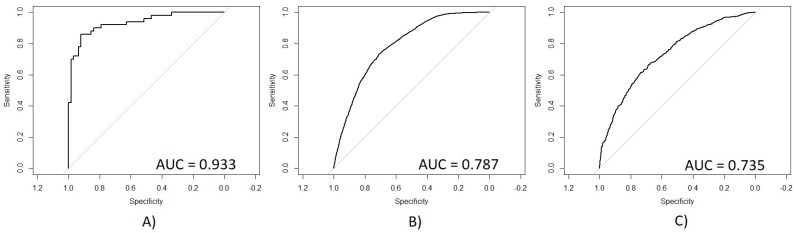
Receiver operating characteristic (ROC) curves of the generated multivariate models for the detection of caries. The horizontal axis represents the 1-specificity measure and the vertical axis represents the sensitivity measure. (**A**) Age group 2, (**B**) Age group 3, (**C**) Age group 4. AUC: area under the curve.

After the validation step based on the NRI parameters, the ROC and AUC parameters were calculated for a second validation. Figure 6 presents in (A) the ROC curve for age group 2, obtaining an AUC value of 0.933. (B) presents the ROC curve for age group 3, obtaining an AUC value of 0.787. (C) presents the result for age group 4, obtaining an AUC value of 0.735.

## 4. Discussion and Conclusions

This section presents the discussion and conclusions for the reported results obtained for this case–control study, where the main objective was to find a multivariate model that allows for classification of subjects with the presence of caries from subjects with their absence, according to their age, looking for the demographic and dietary features that bring the most descriptive information for cases and controls. This helps to indicate when a subject is at risk of suffering from dental caries, making it possible to take preventive measures and thus decreasing this public health problem.

The three multivariate models that were obtained for the age groups present different characteristics in the classification of subjects. Some of the features are present in all three models; nevertheless, there are remarkable differences in specific features that were selected for each group, besides the quantity of features that are contained in each. It is important to remark that due to the difference in the number of subjects belonging to each age group, it was necessary to use two different cutoff values in the selection process; nevertheless, the impact of this difference was not significant according to the results obtained, since it did not cause problems of overfitting and the validation remained consistent between the age groups. Therefore, it is possible to compare them.

In Table 2 it is possible to observe that according to the OR values obtained, most features in the model were statistically significant and the proportion of demographic features was very similar to the proportion of dietary features (15 features and 18 features, respectively), which means that for age group 2, both types of features provided important information in very similar proportion for the classification of subjects. On the other hand, the values obtained from the NRI analysis showed that the reclassification of subjects through the developed multivariate model had a mean proportion of −0.080, which means that the classification was 0.080 better if the standard model (all features) was used to classify cases instead of the new model obtained trough the feature selection. The correct reclassification of subjects with presence of caries had a proportion of 0.040, while the incorrect had a proportion of 0.120. The correct reclassification of control subjects had a proportion of 0.048, which is the same as that obtained for the incorrect reclassification. These values are very significant considering that the reclassification was performed using 33 features instead of 56. In Table 3 it is easier to observe that the reclassification presented a confusion problem to classify subjects in the upper cutoff (i.e., the cases) subjects, showing a higher error in that proportion in comparison with the reclassification proportion of the lower cutoff (i.e., the controls) subjects, being evident in the reclassification of cases subjects exclusively, which means that the features that were selected for this age group may be presenting similar values for some subjects in both type of outcomes, case and control, inducing a classification error in the new model. Figure 3 is a graphical represenation indicating that most subjects were classified in a similar way for both models (standard and new), according to the linear behavior that is shown. Nevertheless, the classification of case and control subjects in the cutoff region can be wrong because it is the range of values where the threshold between both outcomes is present, which means that the subjects that are part of this range of values may belong to controls with a similar probability of belonging to cases.

For age group 3, in Table 4 it is possible to observe that according to the OR values obtained, all features presented a probability of subjects being case very similar to them being control, which means that any feature provided more information to classify cases than the others. The proportion of demographic features was reduced in comparison to the proportion of dietary features (6 demographic features and 12 dietary features), which means that for age group 3, there were more dietary features that provided information for the classification of subjects than demographic fetures. On the other hand, the values obtained from the NRI analysis show that the reclassification of subjects through the developed multivariate model had a mean proportion of −0.024, which means that the classification was 0.024 better if the standard model (all features) was used instead of the new model. From this mean value, 0.019 proportion belongs to the classification of cases and 0.005 proportion belongs to controls; this value is not relatively significant in comparison with the NRI values obtained for age group 2. The correct reclassification of subjects with presence of caries had a proportion of 0.055, while the incorrect had a proportion of 0.074. The correct reclassification of control subjects had a proportion of 0.055, while the incorrect had a proportion of 0.060. Based on these values, it is possible to say that for this new model is was easier to classify control subjects than case. However, the increase of the error in the classification of case subjects is not very significant, considering that the reclassification was performed using 18 features instead of 104. In Table 5 it is easier to observe that the reclassification presented a small proportion of false positives and false negatives; however, the cutoff range presented a significant value of uncertainty due to the high number of subjects that were between the threshold values, which means that the features that were extracted for this age group may be presenting similar values for some subjects in both types of outcome, inducing an error in the classification through the new model. Figure 4 allows graphical observation that most subjects were classified similarly for both models (standard and new), according to the linear behavior that is shown. Nevertheless, the classification of case and control subjects in the cutoff region can be wrong because it is the range of values where subjects may belong to controls with a similar probability of belonging to cases.

Finally, for age group 4, in Table 6 it is possible to observe that according to the OR values obtained, all features presented a probability of subjects being case very similar to the probability of them being control, as in age group 3. This multivariate model presented the smallest number of features in comparison with age groups 2 and 3, and the proportion of demographic features was very similar to the proportion of dietary features (four demographic features and six dietary features), which means that for this age group, both types of features provided important information and in very similar proportion for the classification of subjects. The statistical values obtained from the NRI analysis show that the reclassification of subjects through the developed multivariate model had a mean proportion of −0.129, which means that the classification was 0.129 better if the standard model (all features) was used instead of the new model, being the highest value in the three age groups, where the standard model classified with a better behavior than the new one. This may occur due to the small number of features that are part of this age group. The correct reclassification of subjects with the presence of caries had a proportion of 0.083, while the incorrect had a proportion of 0.096. The correct reclassification of control subjects had a proportion of 0.081, while the incorrect had a proportion of 0.197. Based on these values it is possible to say that for this new model it is easier to classify control subjects than case; however, the increase of the error in the classification of case subjects was not very significant considering that the reclassification was performed using 10 features instead of 104. In Table 7 it is easier to observe that the reclassification presented a very similar proportion of false positives and false negatives. The lower cutoff (i.e., the controls) presented the highest error in the reclassification, and the subjects that were located in the cutoff range also presented a significant value, which means that the features that were extracted for this age group may be presenting similar values for some subjects in both type of outcomes, inducing an error in the classification through the new model. Figure 5 demonstrates that most subjects were classified similarly for both models (standard and new), according to the linear behavior that is shown. Nevertheless, the classification of case and control subjects in the cutoff region can be wrong because it is the range of values where subjects may belong to controls with a similar probability of belonging to cases, besides being remarkable that a significant number of control subjects appeared in those values that would be assigned to case subjects, causing the uncertainty value due to the false positives.

According to the NRI values obtained using the new models, all of them presented statistically significant reclassification values, obtaining parallel proportions of true positives/true negatives, considering that the number of subjects was very different for each age group, as the number of features for each model.

It is important to remark that age group 2 contained the smallest number of subjects but the largest number of features, which means that the smaller the quantity of data, the more difficult it is to generate a general model that correctly classify them, making it necessary to use more features.

On the other hand, Figure 6 presents the ROC obtained for each age group with their respective AUC values. (A) shows a very significant ROC with a sensitivity/specificity proportion of 0.933, which means that 93.3% of subjects from age group 2 were correctly classified using the new model. (B) shows the ROC curve for age group 3 with a sensitivity/specificity proportion of 0.787, which means that 78.7% of the total subjects were correctly classified using the new model. (C) shows the ROC curve for age group 4 with a sensitivity/specificity proportion of 0.735, which means that 73.5% of the subjects were correctly classified. According to these results, it is possible to observe that age group 2 had a remarkably better performance than the other two age groups, which presented very similar AUC values. The accuracy of age group 2 may be reached because this group had a smaller number of subjects and a higher number of features on its multivariate model. However, this relationship of subjects and features may cause an overfit problem, making difficult to generalize this multivariate model. On the other hand, the AUC values obtained for age groups 3 and 4 were also statistically significant, which means that these models presented a good performance in calculating the correct outcome for each subject using the smallest number of data possible, extracted from a very large quantity of data, which means that age groups 3 and 4 did not present the problem of overfitting like age group 2.

According to these results, it is possible to conclude that the use of FBS for feature selection presents a good performance, based on the statistical validation using NRI, ROC, AUC, and OR. The feature selection was used to develop three different multivariate models for the classification of control and case subjects, taking into account the age groups to which the patients belonged in order to know if the age of subjects changed the risk of suffering caries, based on their demographic and dietary information.

All multivariate models that were developed were significant, and each presented different characteristics from the others. The model of age group 2 presented the highest accuracy in the classification of subjects and the proportion of demographic and dietary features was very similar, which means that for subjects that were between 10 and 19 years old (age group 2), both types of features influenced the development of caries. This may occur because at this age range, aside from the importance of the good feeding (information that is present in dietary features), it is important to teach or educate children ranging from 10 years old to teenagers to have good oral health, taking into account the environment in which they are developed (information that is present in demographic features).

For age group 3 a model was developed that presented double the number of dietary features than demographic, which means that subjects between 20 and 59 years old (age group 3) need the information of dietary features more than demographic features to know their dental status and their risk of developing dental caries. Finally, for age group 4, it was more important for subjects that are 60 years old or more (age group 4) to pay more attention in their feeding than in their demographic status in order to avoid caries problems.

Through this statistical analysis it was possible to find which demographic and dietary features were the most significant to bring information about the development of caries in three different groups of people, being a case/control study. These groups of people correspond to three different age ranges. Nevertheless, it is important to consider that one of the main limitations of these results is the type of subjects that were used for the statistical analysis and the number of subjects in the datasets. These databases collected a series of data from a great diversity of subjects, which may represent a general view of the public health problem, but for a centralization of the problem, it would be important to use a more specific dataset with the demographic and dietary features of the population under study (i.e., Mexican subjects). On the other hand, the number of subjects corresponding to each age group is unbalanced, which may induce a bias in the feature selection and the validation process. Another limitation is presented for age groups 3 and 4, which obtained an uncertainty value of around 30%, representing a relative significant error for the prediction of dental caries. Finally, even when the most accurate model was obtained using the data from age group 2, it is important to note that in this age range (10–19 years), those subjects that are younger are dependent on their relatives for their demographic and dietary circumstances, making dental caries reduction not an exclusive problem of the subjects of study, but the environment that surrounds them.

## 5. Future Work

As future work we propose a different feature selection approach for the development of models based on the genetic algorithm Galgo, validated through a forward selection and a backward elimination process, in order to find the similarities and the differences between those results and the obtained in this work, looking for the improvement of the accuracy in the classification of subjects. Additionally, for the validation stage, a random forest method may be used in order to test the accuracy of the models based on a decision tree approach, ensuring the certainty of the model behavior.

On the other hand, we propose the implementation of an app with the purpose of providing an automated calculation of the probability that a subject presents the risk of developing dental caries, based on a questionnaire with the required information, according to the multivariate model developed. This approach may represent a tool for a preliminary diagnosis that may be available independently of the demographic situation, helping to reduce the high incidence of dental caries.

Finally, it has been proven that the demographic situation can strongly influence the prevalence of many conditions or diseases. Based on this, we propose the analysis of oral health data from exclusively Mexican subjects (Zacatecas state) and the comparison of those results with those obtained in this work, in order to prove the differences and the similitudes between the oral health of both populations, and also to develop a preliminary model for the diagnosis and prognosis of caries for each age group in this specific city.

## Figures and Tables

**Table 1:** Developed age groups where subjects were classified.

Age Group	Age	Subjects	Case/Control
1	0–9	0	0
2	10–19	112	50/62
3	20–59	7603	4551/3052
4	60≤	2097	1521/576

**Table 2:** Odds ratio (OR) values and confidence intervals (2.5%/97.5%) obtained by the statistical analysis of the multivariate model corresponding to age group 2, containing 15 demographic features and 18 dietary features. PSU: primary sampling unit.

Age Group 2: 112 Subjects (50 Cases/62 Control), 1.14% of the Total Subjects.				
Feature	Description	OR	2.5%	97.5%
Demographic features				
RIAGENDR	Gender of the participant.	6.269	1.0817	50.844
RIDRETH1	Recode of reported race and Hispanic origin information.	4.17 $\times \;10^{-2}$	2.66 $\times \;10^{-3}$	0.425
RIDRETH3	Recode of reported race and Hispanic origin information, with Non-Hispanic Asian category.	8.391	1.458	63.056
RIDEXMON	Six-month time period when the examination was performed.	1.00 $\times \;10^{2}$	7.763	2967.384
RIDEXAGM	Age in months of the participant at the time of examination.	1.045	1.007	1.093
DMDEDUC3	Highest grade or level of school completed or the highest degree received.	5.07 $\times \;10^{-1}$	2.49 $\times \;10^{-1}$	0.888
DMDMARTL	Marital status.	2.243	9.76 $\times \;10^{-1}$	5.793
DMDFMSIZ	Total number of people in the family.	2.03 $\times \;10^{-1}$	5.38 $\times \;10^{-2}$	0.562
DMDHHSZA	Number of children aged 5 years or younger in the household (HH).	3.640	6.62 $\times \;10^{-1}$	24.505
DMDHRGND	HH reference person’s gender.	2.56 $\times \;10^{-1}$	2.63 $\times \;10^{-2}$	1.842
DMDHREDU	HH reference person’s education level.	8.235	1.962	47.976
SDMVPSU	Masked variance unit pseudo-PSU variable for variance estimation.	6.12 $\times \;10^{-2}$	2.49 $\times \;10^{-3}$	0.864
INDHHIN2	Total household income (reported as a range value in dollars).	9.44 $\times \;10^{-1}$	8.91 $\times \;10^{-1}$	0.990
INDFMIN2	Total family income (reported as a range value in dollars).	1.151	1.023	NA
INDFMPIR	A ratio of family income to poverty guidelines.	4.259	4.60 $\times \;10^{-1}$	40.677
Dietary features				
WTDRD1	Dietary day one sample weight.	1.000	1.000	1.000
WTDR2D	Dietary two-day sample weight.	9.99 $\times \;10^{-1}$	9.99 $\times \;10^{-1}$	1.000
DR1DRSTZ	Dietary recall status.	4.972	1.721	17.311
DBQ095Z	Type of salt usually added to food at the table.	1.102	1.006	NA
DBD100	Frequency with which ordinary salt is added to the food on the table.	2.87 $\times \;10^{-1}$	6.86 $\times \;10^{-2}$	1.003
DRQSPREP	Frequency with which ordinary salt or seasoned salt is added in cooking or preparing foods in the household.	1.612	6.37 $\times \;10^{-1}$	4.444
DR1TKCAL	Energy (kcal).	9.81 $\times \;10^{-1}$	9.54 $\times \;10^{-1}$	1.005
DR1TPROT	Protein (g).	1.138	1.010	1.311
DR1TCARB	Carbohydrate (g).	1.129	1.005	1.298
DR1TSUGR	Total sugars (g).	9.39 $\times \;10^{-1}$	8.82 $\times \;10^{-1}$	0.986
DR1TFIBE	Dietary fiber (g).	6.95 $\times \;10^{-1}$	5.01 $\times \;10^{-1}$	0.903
DR1TTFAT	Total fat (g).	4.78 $\times \;10^{-1}$	2.28 $\times \;10^{-1}$	0.859
DR1TSFAT	Total saturated fatty acids (g).	3.168	1.548	7.820
DR1TMFAT	Total monounsaturated fatty acids (g).	2.409	1.228	5.476
DR1TPFAT	Total polyunsaturated fatty acids (g).	2.349	1.235	5.126
DR1TLYCO	Lycopene (mcg).	1.000	9.99 $\times \;10^{-1}$	1.000
DR1TFA	Folic acid (mcg).	9.74 $\times \;10^{-1}$	9.54 $\times \;10^{-1}$	0.991
DR1TB12A	Added vitamin B12 (mcg).	2.469	1.244	5.555

**Table 3:** Reclassification table obtained for model 2 using NRI, 0.5 and 0.7 values represent the cutoff range used to calculate the risk category. Values in red represent true negatives, values in green represent true positives, and values in the cutoff range are presented in gray. False positives and false negatives are presented in black.

Age Group 2: 112 Subjects (50 Cases/62 Control), 1.14% of the Total Subjects.											
All Subjects				Case				Control			
New				New				New			
Standard	<0.5	<0.7	≥0.7	Standard	<0.5	<0.7	≥0.7	Standard	<0.5	<0.7	≥0.7
<0.5	57	1	1	<0.5	4	0	0	<0.5	53	1	1
<0.7	4	6	3	<0.7	2	4	2	<0.7	2	2	1
≥0.7	1	4	35	≥0.7	1	3	34	≥0.7	0	1	1

**Table 4:** OR values and confidence intervals (2.5%/97.5%) obtained by the statistical analysis of the multivariate model corresponding to age group 3, containing 6 demographic features and 12 dietary features.

Age Group 3: 7603 Subjects (4551 Cases/3052 Control), 77.48% of the Total Subjects.				
Feature	Description	OR	2.5%	97.5%
Demographic features				
RIDAGEYR	Age in years of the participant at the time of screening.	1.029	1.025	1.033
RIDRETH1	Recode of reported race and Hispanic origin information.	0.915	0.876	0.956
RIDEXAGM	Age in months of the participant at the time of examination.	1.006	1.005	1.006
DMDEDUC2	Highest grade or level of school completed or the highest degree received.	1.340	1.270	1.414
DMDHHSZB	Number of children aged 6–17 years old in the household (HH).	1.128	1.076	1.183
DMDHSEDU	HH reference person’s spouse’s education level.	0.837	0.789	0.888
Dietary features				
WTDR2D	Dietary two-day sample weight.	1.000	1.000	1.000
DR1DRSTZ	Dietary recall status.	0.923	0.886	0.962
DR1TKCAL	Energy (kcal).	0.992	0.989	0.996
DR1TPROT	Protein (g).	1.031	1.017	1.046
DR1TCARB	Carbohydrate (g).	1.029	1.016	1.042
DR1TTFAT	Total fat (g).	1.071	1.040	1.103
DR1TCHOL	Cholesterol (mg).	1.001	1.000	1.001
DR1TFA	Folic acid (mcg).	1.001	1.000	1.001
DR1TCHL	Total choline (mg).	0.998	0.997	0.999
DR1TIRON	Iron (mg).	0.969	0.957	0.982
DR1TALCO	Alcohol (g).	1.062	1.038	1.088
DR1TS080	SFA 8:0 (Octanoic) (g).	0.564	0.445	0.708

**Table 5:** Reclassification table obtained for model 3 using NRI; 0.5 and 0.7 values represent the cutoff range used to calculate the risk category. Values in red represent true negatives, values in green represent true positives, and values in the cutoff range are presented in gray. False positives and false negatives are presented in black.

Age Group 3: 7603 Subjects (4551 Cases/3052 Control), 77.48% of the Total Subjects.											
All Subjects				Case				Control			
New				New				New			
Standard	<0.5	<0.7	≥0.7	Standard	<0.5	<0.7	≥0.7	Standard	<0.5	<0.7	≥0.7
<0.5	2244	195	8	<0.5	610	83	5	<0.5	1634	112	3
<0.7	223	1252	236	<0.7	124	745	166	<0.7	99	507	70
≥0.7	3	284	3158	≥0.7	3	214	2601	≥0.7	0	70	557

**Table 6:** OR values and confidence intervals (2.5%/97.5%) obtained by the statistical analysis of the multivariate model corresponding to age group 4, containing four demographic features and six dietary features.

Age Group 4: 2097 Subjects (1521 Cases/576 Control), 21.37% of the Total Subjects.				
Feature	Description	OR	2.5%	97.5%
Demographic features				
RIDRETH1	Recode of reported race and Hispanic origin information.	0.777	0.704	0.856
RIDEXAGM	Age in months of the participant at the time of examination.	1.004	1.003	1.006
DMDEDUC2	Highest grade or level of school completed or the highest degree received.	1.471	1.339	1.618
INDFMPIR	Ratio of family income to poverty guidelines.	1.205	1.112	1.308
Dietary features				
DR1DRSTZ	Dietary recall status.	0.770	0.712	0.833
DR1TATOC	Vitamin E as alpha-tocopherol (mg).	1.087	1.049	1.128
DR1TATOA	Added alpha-tocopherol (Vitamin E) (mg).	0.909	0.870	0.951
DR1TVD	Vitamin D (D2 + D3) (mcg).	0.958	0.938	0.980
DR1TPHOS	Phosphorus (mg).	1.000	1.000	1.000
DR1TS160	SFA 16:0 (Hexadecanoic) (g).	0.943	0.921	0.964

**Table 7:** Reclassification table obtained for model 4 using NRI, 0.5 and 0.7 values represent the cutoff range used to calculate the risk category. Values in red represent true negatives, values in green represent true positives, and values in the cutoff range are presented in gray. False positives and false negatives are presented in black.

All Subjects				Case				Control			
New				New				New			
Standard	<0.5	<0.7	≥0.7	Standard	<0.5	<0.7	≥0.7	Standard	<0.5	<0.7	≥0.7
<0.5	193	81	16	<0.5	65	33	7	<0.5	28	48	9
<0.7	42	299	144	<0.7	24	177	87	<0.7	18	122	57
≥0.7	2	150	1170	≥0.7	1	122	1005	≥0.7	1	28	165

## Appendix A

### Appendix A.1.

**Table A1:** Description of demographic features.

Feature	Description
AIALANGA	Language of the MEC ACASI Interview Instrument.
DMDBORN4	In what country was Sample Person (SP) born?
DMDCITZN	Is SP a citizen of the United States?
DMDHHSIZ	Total number of people in the household.
DMDHHSZE	Number of adults aged 60 years or older in the household.
DMDEDUC2	Highest grade or level of school completed or the highest degree received.
DMDHHSZB	Number of children aged 6–17 years old in the household (HH).
DMDHSEDU	HH reference person’s spouse’s education level.
DMDMARTL	Marital status.
DMDFMSIZ	Total number of people in the Family.
DMDHHSZA	Number of children aged 5 years or younger in the household.
DMDHRGND	HH reference person’s gender.
DMDHREDU	HH reference person’s education level.
DMDEDUC3	Highest grade or level of school completed or the highest degree received.
DMDHRAGE	HH reference person’s age in years.
DMDHRBR4	HH reference person’s country of birth.
DMDHREDU	HH reference person’s education level.
DMDHRMAR	HH reference person’s marital status.
DMDYRSUS	Length of time the participant has been in the US.
DMQADFC	Did SP ever serve in a foreign country during a time of armed conflict or on a humanitarian or peace-keeping mission?
DMQMILIZ	Has SP ever served on active duty in the U.S. Armed Forces, military Reserves, or National Guard?
FIAINTRP	Was an interpreter used to conduct the Family interview?
FIALANG	Language of the Family Interview Instrument.
FIAPROXY	Was a Proxy respondent used in conducting the Family Interview?
INDFMPIR	A ratio of family income to poverty guidelines.
INDHHIN2	Total household income (reported as a range value in dollars).
INDFMIN2	Total family income (reported as a range value in dollars).
LATVPSU	Variance unit: PSU variable for variance estimation.
LATVSTRA	Variance unit: stratum variable for variance estimation.
MIAINTRP	Was an interpreter used to conduct the MEC CAPI interview?
MIALANG	Language of the MEC CAPI Interview Instrument.
MIAPROXY	Was a Proxy respondent used in conducting the MEC CAPI Interview?
RIAGENDR	Gender of the participant.
RIDAGEEX	Age in years of the participant at the time of examination. Individuals aged 959 months and older are topcoded at 959 months.
RIDAGEMN	ge in months of the participant at the time of screening. Reported for persons aged 24 months or younger at the time of exam.
RIDRETH3	Recode of reported race and Hispanic origin information, with Non-Hispanic Asian Category.
RIDEXMON	Six month time period when the examination was performed.
RIDEXPRG	Pregnancy status for females between 20 and 44 years of age at the time of MEC exam.
RIDAGEYR	Age in years of the participant at the time of screening.
RIDRETH1	Recode of reported race and Hispanic origin information.
RIDEXAGM	Age in months of the participant at the time of examination.
RIDSTATR	Interview and examination status of the participant.
SDMVPSU	Masked variance unit pseudo-PSU variable for variance estimation.
SDDSRVYR	Data release cycle.

**Table A2:** Description of demographic features.

Feature	Description
SDMVSTRA	Masked variance unit pseudo-stratum variable for variance estimation.
WTLAF8YR	Subsample 8-year fasting weight for participants aged 12 years and older who were examined in the morning sessions in Los Angeles, California.
WTLAI8YR	Full sample 8-year interview weight for participants in Los Angeles, California.
WTLAM8YR	Full sample 8-year MEC exam weight for participants in Los Angeles, California.

### Appendix A.2.

**Table A3:** Description of dietary features.

Feature	Description
WTDR2D	Dietary two-day sample weight.
DR1DRSTZ	Dietary recall status.
DR1TPROT	Protein (g).
DR1TCARB	Carbohydrate (g).
DR1TTFAT	Total fat (g).
DR1TCHOL	Cholesterol (mg).
DR1TFA	Folic acid (mcg).
DR1TCHL	Total choline (mg).
DR1TIRON	Iron (mg).
DR1TALCO	Alcohol (g).
DR1TS080	SFA 8:0 (Octanoic) (g).
DR1TATOC	Vitamin E as alpha-tocopherol (mg).
DR1TATOA	Added alpha-tocopherol (Vitamin E) (mg).
DR1TVD	Vitamin D (D2 + D3) (mcg).
DR1TPHOS	Phosphorus (mg).
DR1TS160	SFA 16:0 (Hexadecanoic) (g).
WTDRD1	Dietary day one sample weight.
DBQ095Z	Type of salt usually add to food at the table.
DBD100	Frequency with which ordinary salt is added to the food on the table.
DRQSPREP	Frequency with which ordinary salt or seasoned salt is added in cooking or preparing foods in the household.
DR1TKCAL	Energy (kcal).
DR1TSUGR	Total sugars (g).
DR1TFIBE	Dietary fiber (g).
DR1TSFAT	Total saturated fatty acids (g).
DR1TMFAT	Total monounsaturated fatty acids (g).
DR1TPFAT	Total polyunsaturated fatty acids (g).
DR1TLYCO	Lycopene (mcg).
DR1TFA	Folic acid (mcg).
DR1TB12A	Added vitamin B12 (mcg).
DR1_300	Was the amount of food that you ate yesterday much more than usual, usual, or much less than usual?
DR1_320Z	Total plain water drank yesterday—including plain tap water, water from a drinking fountain, among others.
DR1_330Z	Total tap water drank yesterday—including filtered tap water and water from a drinking fountain.
DR1BWATZ	Total bottled water drank yesterday (g).
DR1DAY	Intake day of the week.
DR1DBIH	Number of days between intake day and the day of family questionnaire administered in the household.
DR1SKY	What type of salt was it? (Was it ordinary or seasoned salt, lite salt, or a salt substitute?).
DR1STY	Did SP add any salt to her/his food at the table yesterday?
DR1TACAR	Alpha-carotene (mcg).
DR1TBCAR	Beta-carotene (mcg).
DR1TCAFF	Caffeine (mg).
DR1TCALC	Calcium (mg).
DR1TCOPP	Copper (mg).
DR1TCRYP	Beta-cryptoxanthin (mcg).
DR1TFDFE	Folate as dietary folate equivalents (mcg).
DR1TFF	Food folate (mcg).
DR1TFOLA	Total folate (mcg).
DR1TLZ	Lutein + zeaxanthin (mcg).
DR1TM161	MFA 16:1 (Hexadecenoic) (g).
DR1TM181	MFA 18:1 (Octadecenoic) (g).
DR1TM201	MFA 20:1 (Eicosenoic) (g).

**Table A4:** Description of dietary features.

Feature	Description
DR1TM221	MFA 22:1 (Docosenoic) (g).
DR1TMAGN	Magnesium (mg).
DR1TMOIS	Moisture (g).
DR1TNIAC	Niacin (mg).
DR1TNUMF	Total number of foods/beverages reported in the individual foods file.
DR1TP182	PFA 18:2 (Octadecadienoic) (g).
DR1TP183	PFA 18:3 (Octadecatrienoic) (g).
DR1TP184	PFA 18:4 (Octadecatetraenoic) (g).
DR1TP204	PFA 20:4 (Eicosatetraenoic) (g).
DR1TP205	PFA 20:5 (Eicosapentaenoic) (g).
DR1TP225	PFA 22:5 (Docosapentaenoic) (g).
DR1TP226	PFA 22:6 (Docosahexaenoic) (g).
DR1TPOTA	Potassium (mg).
DR1TRET	Retinol (mcg).
DR1TS040	SFA 4:0 (Butanoic) (g).
DR1TS060	SFA 6:0 (Hexanoic) (g).
DR1TS100	SFA 10:0 (Decanoic) (g).
DR1TS120	SFA 12:0 (Dodecanoic) (g).
DR1TS140	SFA 14:0 (Tetradecanoic) (g).
DR1TS180	SFA 18:0 (Octadecanoic) (g).
DR1TSELE	Selenium (mcg).
DR1TSODI	Sodium (mg).
DR1TTHEO	Theobromine (mg).
DR1TVARA	Vitamin A as retinol activity equivalents (mcg).
DR1TVB1	Thiamin (Vitamin B1) (mg).
DR1TVB12	Vitamin B12 (mcg).
DR1TVB2	Riboflavin (Vitamin B2) (mg).
DR1TVB6	Vitamin B6 (mg).
DR1TVC	Vitamin C (mg).
DR1TVK	Vitamin K (mcg).
DR1TWS	When you drink tap water, what is the main source of the tap water? Is the city water supply; a well or rain cistern; a spring; or something else?
DR1TZINC	Zinc (mg).
DRABF	Indicates whether the sample person was an infant who was breast-fed on either of the two recall days.
DRD340	During the past 30 days did you eat any types of shellfish listed on this card?
DRD350A	Clams eaten during the past 30 days.
DRD350AQ	Number of times clams were eaten in the past 30 days.
DRD350B	Crabs eaten during the past 30 days.
DRD350BQ	Number of times crab was eaten in the past 30 days.
DRD350C	Crayfish eaten during the past 30 days.
DRD350CQ	Number of times crayfish was eaten in the past 30 days.
DRD350D	Lobsters eaten during the past 30 days.
DRD350DQ	Number of times lobster was eaten in the past 30 days.
DRD350E	Mussels eaten during the past 30 days.
DRD350EQ	Number of times mussels were eaten in the past 30 days.
DRD350F	Oysters eaten during the past 30 days.
DRD350FQ	Number of times oysters were eaten in the past 30 days.
DRD350G	Scallops eaten during the past 30 days.
DRD350GQ	Number of times scallops were eaten in the past 30 days.
DRD350H	Shrimp eaten during the past 30 days.
DRD350HQ	Number of times shrimp was eaten in the last 30 days.
DRD350I	Other shellfish ( ex. octopus, squid) eaten during the past 30 days.
DRD350IQ	Number of times other shellfish (ex. octopus, squid) was eaten in the past 30 days.
DRD350J	Other unknown shellfish eaten during the past 30 days.
DRD350JQ	Number of times other unknown shellfish was eaten in the past 30 days.
DRD350K	Refused to give detailed information on shellfish eaten during the past 30 days.

**Table A5:** Description of dietary features.

Feature	Description
DRD360	During the past 30 days did you eat any types of fish listed on this card?
DRD370A	Breaded fish products eaten during the past 30 days.
DRD370AQ	Number of times breaded fish products were eaten in the past 30 days.
DRD370B	Tuna eaten during the past 30 days.
DRD370BQ	Number of times tuna was eaten in the past 30 days.
DRD370C	Bass eaten during the past 30 days.
DRD370CQ	Number of times bass was eaten in the past 30 days.
DRD370D	Catfish eaten during the past 30 days.
DRD370DQ	Number of times catfish was eaten in the past 30 days.
DRD370E	Cod eaten during the past 30 days.
DRD370EQ	Number of times cod was eaten in the past 30 days.
DRD370F	Flatfish eaten during the past 30 days.
DRD370FQ	Number of times flatfish was eaten in the past 30 days.
DRD370G	Haddock eaten during the past 30 days.
DRD370GQ	Number of times haddock was eaten in the past 30 days.
DRD370H	Mackerel eaten during the past 30 days.
DRD370HQ	Number of times mackerel was eaten in the past 30 days.
DRD370I	Perch eaten during the past 30 days.
DRD370IQ	Number of times perch was eaten in the past 30 days.
DRD370J	Pike eaten during the past 30 days.
DRD370JQ	Number of times pike was eaten in the past 30 days.
DRD370K	Pollock eaten during the past 30 days.
DRD370KQ	Number of times pollock was eaten in the past 30 days.
DRD370TQ	Number of times other type of fish was eaten in the past 30 days.
DRD370V	Refused to give detailed information on fish eaten during the past 30 days.
DRQSDIET	Are you currently on any kind of diet, either to lose weight or for some other health-related reason?
DRQSDT1	What kind of diet are you on? (Is it a weight loss or low calorie diet: low fat or cholesterol diet; low salt or sodium diet; sugar free or low sugar diet; low fiber diet; high fiber diet; diabetic diet; or another type of diet?).
DRD370L	Porgy eaten during the past 30 days.
DRD370LQ	Number of times porgy was eaten in the past 30 days.
DRD370M	Salmon eaten during the past 30 days.
DRD370MQ	Number of times salmon was eaten in the past 30 days.
DRD370N	Sardines eaten during the past 30 days.
DRD370NQ	Number of times sardines were eaten in the past 30 days.
DRD370O	Sea bass eaten during the past 30 days.
DRD370OQ	Number of times sea bass was eaten in the past 30 days.
DRD370U	Other unknown type eaten during the past 30 days.
